# Characteristics of road traffic mortality and distribution of healthcare resources in Thailand

**DOI:** 10.1038/s41598-022-24811-4

**Published:** 2022-11-24

**Authors:** Kasem Seresirikachorn, Panisa Singhanetr, Ngamphol Soonthornworasiri, Anyarak Amornpetchsathaporn, Thanaruk Theeramunkong

**Affiliations:** 1 Sirindhorn International Institute of Technology, Thammasat University, Pathumthani, Thailand; 2 Mettapracharak Eye Institute, Mettapracharak (Wat Rai Khing) Hospital, Nakhon Pathom, Thailand; 3 Department of Tropical Hygiene, Faculty of Tropical Medicine, Mahidol University, Bangkok, Thailand; 4 Department of Ophthalmology, College of Medicine, Rajavithi Hospital, Rangsit University, Bangkok, Thailand

**Keywords:** Health care, Risk factors

## Abstract

Road traffic mortalities (RTMs), a leading cause of death globally, mostly occur in low- and middle-income countries, and having sufficient healthcare resources could lower the number of these fatalities. Our study aimed to illustrate the incidence of RTMs per 100,000 population and to compare the distribution of healthcare resources from 2011 to 2021 with rates of RTMs in the 77 provinces of Thailand. We divided the population into adults (≥ 15 years) and children (0–14 years). Lorenz curve and Gini coefficient were used to measure the level of distribution and equality of hospital resources and in relation to RTMs across the country. The average number of deaths was 30.34 per 100,000 per year, with male predominance. The RTM rates for adults and children were 32.71 and 19.08 per 100,000 respectively, and motorcycle accidents accounted for the largest percentage of deaths across all age groups. The Gini coefficient showed that operating rooms (0.42) were the least equally distributed hospital resource, while physicians were the most equally distributed (0.34). Anomalies were identified between the distribution of RTMs and available hospital resources. We hope our study will be beneficial in reallocating these resources more fairly to reflect the different numbers of traffic accidents in each province with the aim of reducing lower traffic-related deaths.

## Introduction

Road traffic injuries (RTIs) are among the leading causes of death, and it is expected that by 2030 they will rise from 9 to 7th in the global ranking, causing significant public health concerns^1^^,^^2^. A road traffic mortality (RTM) is defined as any person killed immediately or dying within 30 days of a road traffic incident^3^. Ninety percent of these deaths occur in low- and middle-income countries (LMICs) despite the fact that these nations have only 54% of the vehicles in the world^4^. According to the World Health Organisation (WHO) estimates, there are 32.7 fatalities per 100,000 people, with motorcycles accounting for the majority most of these deaths^5^. Traffic-related mortality and morbidity in LMICs have created a great burden, with an estimated 90% of disability-adjusted life years (DALYs) being lost worldwide as a result of RTIs which occur in LMICs^6^.

Pre-hospital and in-hospital trauma services for LMICs have been found to be insufficient^7,8^. Regions with low and moderate health care resources (HCRs) have been found to have higher rates of mortality from RTIs than those with high HCRs^9^. Hence, if HCRs were re-distributed to cater for regions with high rates of RTMs, the RTI mortality and morbidity rates could potentially be reduced.

Thailand, a middle-income country, had the second-highest rate of RTMs globally in 2015^5^, which was partly due to a sharp rise in motorisation triggered by economic expansion^6^. In 2017, road accidents cost Thailand over 60 million dollars, approximately 0.8% of the nation’s gross domestic product (GDP)^10^.

More than half of all road traffic deaths are amongst vulnerable road users such as pedestrians, cyclists, and motorcyclists. Male victims have been reported to outnumber females approximately threefold, with motorcyclists accounting for about four-fifths of fatalities^11^. Globally, 186,300 children aged between 0 and 9 years, die from road traffic accidents each year^12^. Injured children who are treated at paediatric trauma centres have better outcomes than those who are cared for in an adult centre or a non-trauma centre^13^. This reflects the fact that children require specific medical care which is different from that needed for adults; therefore, statistics on children’s mortality rates need to be investigated.

This report aims to assess the equality of the distribution of hospital resources, taking into account RTMs in Thailand; to observe RTM trends over 11 years for the whole population, dealing with adults and children separately; and to analyse potential factors affecting RTM rates.

## Methods

### Study setting

This study gathered information from Thailand, a middle-income country located in Southeast Asia populated by over 70 million people across 513,120 km^2^. There are 77 provinces, and Bangkok is the capital city. The total number of registered vehicles in 2022 was 41,346,378, of which half were motorcycles.

This retrospective, cross-sectional study was approved by the institutional review board (IRB) of Rajavithi Hospital, Thailand and was conducted in accordance with the tenets of the Declaration of Helsinki. Informed consent was not obtained in this study, as approved by the hospital’s IRB.

The number of RTMs were recorded over an 11-year period between January 1, 2011 and December 31, 2021. The RTM data were compiled by the Department of Disease Control of the Ministry of Public Health, which gathered details from three sources: death certificates; reported deaths from traffic injuries on the Police Information System; and from the central motor vehicle insurance registry. All data were approved and released on the official website of the Digital Government Development Agency.

Data included age, sex, type of accident (motorcycles, cars, pedestrians, trucks including ten wheelers and agriculture vehicles, and bicycles), and cause of death. The latter was recorded using the 10th revision of the International Statistical Classification of Diseases and Related Health Problems (ICD10).

The National Statistical Office provided information regarding Thailand’s population and healthcare personnel and facilities. The population was divided into adults and children, defined according to Thailand’s law as those aged below 15 years. The total population was taken as the average population over 11 years. In terms of hospital resources, we included number of hospital beds, operating rooms (ORs), intensive care unit (ICU) beds, physicians, and nurses. Information regarding hospital resources were gathered for each province over the same 11-year period as the population data.

We also reported RTMs before and during the COVID-19 pandemic, which we defined as the (two-year) pandemic period between January 1st, 2020 and December 31st, 2021.

Factors that were used to assess the correlations with RTMs in the 77 provinces in Thailand were as follows: average income per year; the number of registered vehicles; the volume of oil usage; and the volume of rain, details of which were provided by The National Statistical Office.

### Statistical analysis

Road traffic mortality rates per 100,000 population were calculated and divided into three groups (total, adults, and children) for all provinces. The number of beds, ORs, ICU beds, physicians, and nurses were also calculated per 100,000 population for each province. Frequencies and percentages were used for categorical data while continuous data were reported using mean, median, and standard deviation (SD) after confirmation of normal distribution of the data. Paired-T test was used to compare the number of fatalities during and prior to the COVID-19 pandemic.

Lorenz curve and Gini coefficient were used to measure the level of distribution and equality of health care resources and in relation to incidences of RTMs across the country. Spearman rank-order correlation analysis was used to evaluate the correlations between related factors and RTMs.

The Lorenz curve compares the distribution of a specific variable with uniform distribution representing equality, which is shown by a diagonal line^14,15^. The farther the Lorenz curve is from the diagonal line, the greater the inequality. The cumulative proportion of the RTMs is shown on the x axis, and the cumulative proportion of healthcare resources on the y axis. The Gini coefficient is the ratio of the area between the Lorenz curve and the diagonal line, to the area below the diagonal line in the Lorenz curve. Gini coefficient values range from 0 to 1, with 0 being perfect equality, and 1 representing the maximum inequality possible.

All statistical analyses were done with Microsoft excel and SPSS version 22 for Windows, and a p-value of < 0.05 was consider statistically significant.

### Ethics declarations

This study has been approved by the Ethics Committee of Rajavithi Hospital (Number 65067).

## Results

### Overall

**Figure 1:**
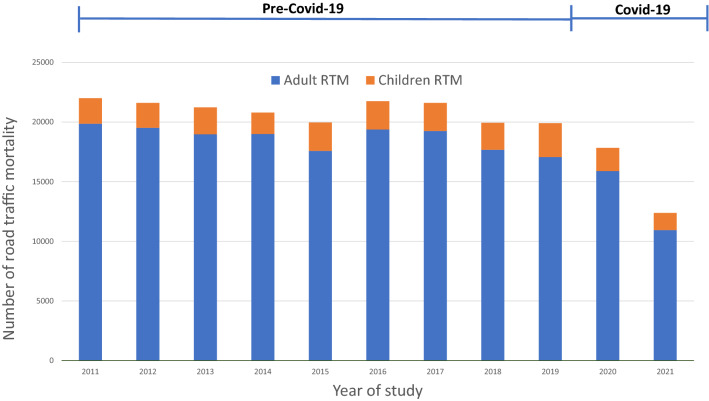
Number of road traffic mortalities from 2011 to 2021 (pre-Covid 19 2011–2019, Covid-19; 2020–2021).

The total number of RTMs over these 11 years was 218,964, of which the majority were adults (89.11%). The average number of deaths was 30.34 per 100,000 people per year with male predominance. The RTI mortality rates for adults and children were 32.71 and 19.08 per 100,000 respectively (Table 1, Fig. 1).

**Table 1:** Population, sex, mean age, and road traffic mortality (death/100,000 population), divided by adults and children during 2011–2021.

Year	Total				Adults				Children			
	Population	Male (%)	Mean age; years (SD)	Death/100,000	Population	Male (%)	Mean age; years (SD)	Death/100,000	Population	Male (%)	Mean age; years (SD)	Death/100,000
2011	64,076,033	67.70	39.74 (19.78)	34.33	51,384,576	63.22	43.87 (16.65)	38.64	12,691,457	65.11	9.16 (3.94)	16.87
2012	64,456,695	81.38	39.92 (19.97)	33.52	52,579,668	80.28	43.61 (16.66)	37.13	11,877,027	81.57	9.82 (3.96)	17.52
2013	64,785,909	78.15	40.15 (20.38)	32.76	53,068,185	76.31	44.65 (18.23)	35.75	11,717,724	79.86	9.63 (5.11)	19.21
2014	65,124,716	69.40	39.18 (18.96)	31.92	53,508,852	66.78	42.66 (19.95)	35.50	11,615,864	65.53	10.34 (4.60)	15.44
2015	65,729,098	83.88	38.96 (19.46)	30.37	54,240,134	85.42	42.58 (18.87)	32.40	11,488,964	82.07	10.94 (3.97)	20.75
2016	65,931,550	84.48	40.35 (19.56)	32.98	54,571,068	83.83	43.82 (18.05)	35.49	11,360,482	84.63	11.32 (3.64)	20.91
2017	66,188,503	69.70	39.99 (19.99)	32.64	54,959,465	67.44	43.82 (18.87)	35.03	11,229,038	71.49	10.61 (3.85)	20.98
2018	66,413,979	89.48	38.58 (19.15)	30.01	55,267,472	87.55	42.48 (17.85)	31.97	11,146,507	86.46	11.73 (4.18)	20.28
2019	66,558,935	75.81	38.52 (22.75)	29.90	55,683,698	77.01	44.18 (18.00)	30.65	10,875,237	75.23	8.19 (4.29)	26.09
2020	66,186,727	94.69	39.99 (18.78)	26.94	55,523,801	95.05	43.32 (17.86)	28.61	10,662,926	91.72	9.78 (4.35)	18.23
2021	66,171,439	91.62	41.06 (20.31)	18.70	55,783,065	93.36	44.94 (17.77)	19.62	10,388,374	89.86	10.07 (4.16)	13.77
Average	65,754,755	79.23	39.68 (19.92)	30.34	54,233,635	80.44	43.49 (17.77)	32.71	11,368,509	76.46	10.45 (4.19)	19.08

A statistically significant difference was found between the overall rates of RTMs before COVID-19 and during the pandemic, with average ratios of 32.05 (± 1.61) and 22.82 (± 5.83) (P = 0.001) deaths per 100,000 respectively.

RTMs for the entire population were highest in 2011, at 34.33 per 100,000 and lowest in 2021 at 18.7 per 100,000. RTMs involving children were highest in 2019, going up to 26.09 deaths per 100,000 children (Table 1).

Overall, the mean age of death was 39.68 ± 19.92 years. With regard to children, the mean age of death was 10.45 ± 4.19 years (Table 1).

### Type of road accidents

Motorcycle accidents were the most common cause of death across all age groups, while bicycle accidents were responsible for less than 1% of RTMs throughout the period. Over these 11 years, the type of accidents showed a relatively similar trend for adults and children (Table 2). The most common ICD-10 diagnosis for the cause of death was V892: person injured in unspecified motor-vehicle accident, at 43.35%, followed by V299: motorcycle rider(driver)(passenger) injured in unspecified traffic accident, at 14.12%.

**Table 2:** The number and percentages of road traffic mortalities (RTM) per year according to type of accident (M; motorcycles, C; cars, P; pedestrians, T;trucks including ten wheelers and agriculture vehicles, and B; bicycles) during 2011–2021.

Year	Total RTM						Adult RTM						Children RTM					
	Overall	M (%)	C (%)	P (%)	T (%)	B (%)	Overall	M (%)	C (%)	P (%)	T (%)	B (%)	Overall	M (%)	C (%)	P (%)	T (%)	B (%)
2011	21,996	14,069 (63.96)	4162 (18.92)	2195 (9.98)	1309 (5.95)	261 (1.19)	19,855	12,590 (63.41)	3861 (19.45)	1974 (9.94)	1201 (6.05)	229 (1.15)	2141	1443 (67.41)	330 (15.41)	224 (10.46)	114 (5.32)	30 (1.40)
2012	21,603	13,477 (62.38)	4498 (20.82)	2001 (9.27)	1435 (6.64)	192 (0.89)	19,521	11,911 (61.02)	4250 (21.77)	1827 (9.36)	1351 (6.92)	182 (0.93)	2082	1466 (70.41)	313 (15.03)	184 (8.84)	104 (5)	15 (0.72)
2013	21,221	12,585 (59.30)	4975 (23.45)	2218 (10.45)	1250 (5.89)	193 (0.91)	18,970	10,950 (57.72)	4689 (24.72)	1977 (10.42)	1178 (6.21)	176 (0.93)	2251	1562 (69.4)	345 (15.33)	240 (10.66)	87 (3.86)	17 (0.75)
2014	20,790	16,546 (79.59)	2219 (10.67)	1195 (5.75)	707 (3.40)	123 (0.59)	18,997	14,935 (78.62)	2135 (11.24)	1098 (5.78)	709 (3.73)	120 (0.63)	1793	1534 (85.58)	127 (7.1)	102 (5.67)	23 (1.29)	7 (0.37)
2015	19,960	16,718 (83.76)	1910 (9.57)	651 (3.26)	579 (2.9)	102 (0.51)	17,576	14,586 (82.99)	1800 (10.24)	578 (3.29)	522 (2.97)	90 (0.51)	2384	2122 (89)	137 (5.76)	81 (3.38)	32 (1.35)	12 (0.51)
2016	21,745	17,500 (80.48)	2914 (13.4)	461 (2.12)	831 (3.82)	39 (0.18)	19,369	15,702 (81.07)	2427 (12.53)	438 (2.26)	761 (3.93)	41 (0.21)	2376	2070 (87.10)	205 (8.64)	36 (1.52)	65 (2.74)	0 (0)
2017	21,607	17,754 (82.17)	1947 (9.01)	767 (3.55)	871 (4.03)	268 (1.24)	19,251	15,702 (81.57)	1833 (9.52)	707 (3.67)	780 (4.05)	229 (1.19)	2356	2085 (88.49)	129 (5.48)	76 (3.23)	48 (2.04)	18 (0.76)
2018	19,931	16,180 (81.18)	1998 (10.02)	1055 (5.29)	608 (3.05)	90 (0.46)	17,671	14,200 (80.36)	1885 (10.67)	946 (5.35)	555 (3.14)	85 (0.48)	2260	1941 (85.88)	143 (6.33)	112 (4.96)	58 (2.56)	6 (0.27)
2019	19,904	13,975 (70.21)	3298 (16.57)	1473 (7.40)	989 (4.97)	169 (0.85)	17,066	11,711 (68.62)	2997 (17.56)	1287 (7.54)	916 (5.37)	155 (0.91)	2838	2266 (79.84)	302 (10.65)	186 (6.55)	70 (2.47)	14 (0.49)
2020	17,831	11,644 (65.30)	3188 (17.88)	1547 (8.68)	1161 (6.51)	291 (1.63)	15,887	10,217 (64.31)	2965 (18.66)	1376 (8.66)	1056 (6.65)	273 (1.72)	1944	1431 (73.61)	221 (11.37)	172 (8.85)	104 (5.35)	16 (0.82)
2021	12,376	7880 (63.66)	2329 (28.82)	1278 (10.32)	689 (5.57)	200 (1.63)	10,946	6879 (62.84)	2157 (19.71)	1087 (9.93)	645 (5.89)	178 (1.63)	1430	994 (69.51)	179 (12.51)	189 (13.22)	46 (3.22)	22 (1.54)
Overall	218,964	158,328 (72.31)	33,438 (15.27)	14,841 (6.78)	10,429 (4.76)	1928 (0.88)	195,109	139,383 (71.44)	30,999 (15.89)	13,295 (6.81)	9674 (4.96)	1758 (0.90)	23,855	18,914 (79.28)	2431 (10.19)	1602 (6.72)	751 (3.15)	157 (0.66)

### RTMs across each province

**Figure 2:**
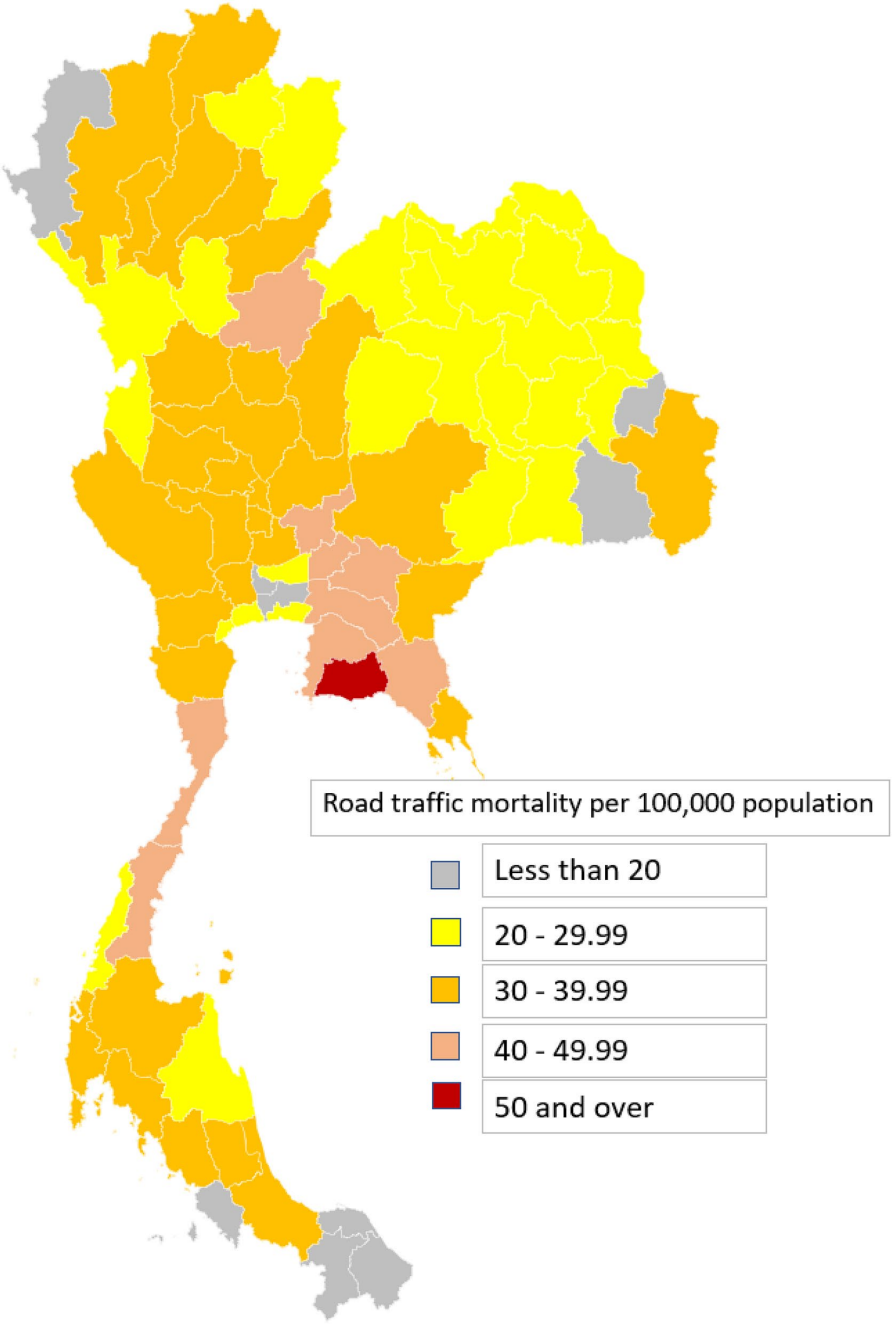
Heat map of Thailand illustrating the number of road traffic mortalities per 100,000 population in all 77 provinces from 2011 to 2021.

Rayong had the highest number of RTMs for the whole population and for adults at 62.05 and 66.94 deaths per 100,000 population respectively while Mae Hong Sorn had the fewest RTMs per 100,000 population out of all 77 provinces. Bangkok, the capital city of Thailand, had 14.41 RTMs per 100,000 population, ranking it as the third-lowest province in terms of RTMs per population (Supplementary Table S1). Rates of RTMs per 100,000 population are illustrated on the heat map (Fig. 2).

**Table 3:** Gini coefficients for hospital resources and road traffic mortalities during 2011–2021.

Hospital resources	Gini coefficients
Hospital beds	0.39
Operating rooms	0.42
Intensive care unit (ICU) beds	0.38
Physicians	0.34
Nurses	0.37

### Distribution of hospital resources

Bangkok had the most hospital resources per 100,000 population in terms of hospital beds, ORs, ICU beds, physicians, and nurses. The average number of hospital beds and ICUs were 164.11 and 9.46 per 100,000 population respectively. Pathum Thani had the lowest number of beds and ORs per population, while the lowest number of ICUs per population was found in Chaiyaphum. There were on average 37.34 physicians and 207.65 nurses per 100,000 people. Samut Sakhon had the lowest number of physicians, while Bueng Kan had the fewest nurses (Supplementary Table S1).

**Figure 3:**
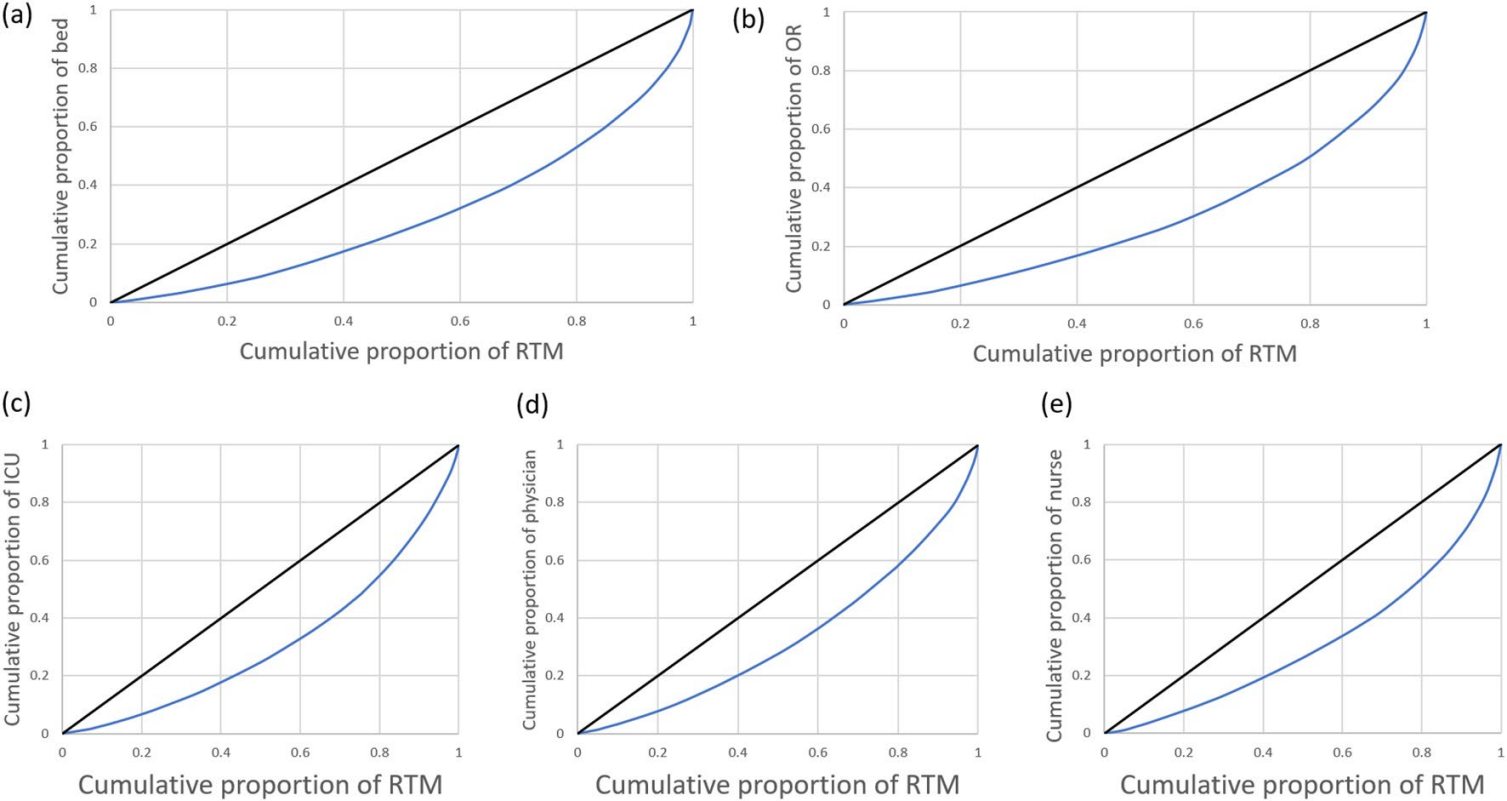
Lorenz curves illustrating equality in distribution of hospital resources and road traffic mortalities (RTMs); (**a**) hospital beds(bed), (**b**) operating rooms (ORs), (**c**) intensive care unit (ICU) beds, (**d**) physicians, and (**e**) nurses between 2011 and 2021.

The levels of equality in distribution of HCRs throughout Thailand are illustrated by Lorenz curve (Fig. 3). According to the Gini coefficient, the resource that was least equally distributed was the number of ORs (0.42), while physicians were most equally distributed (0.34) (Table 3).

### Associated factors

**Figure 4:**
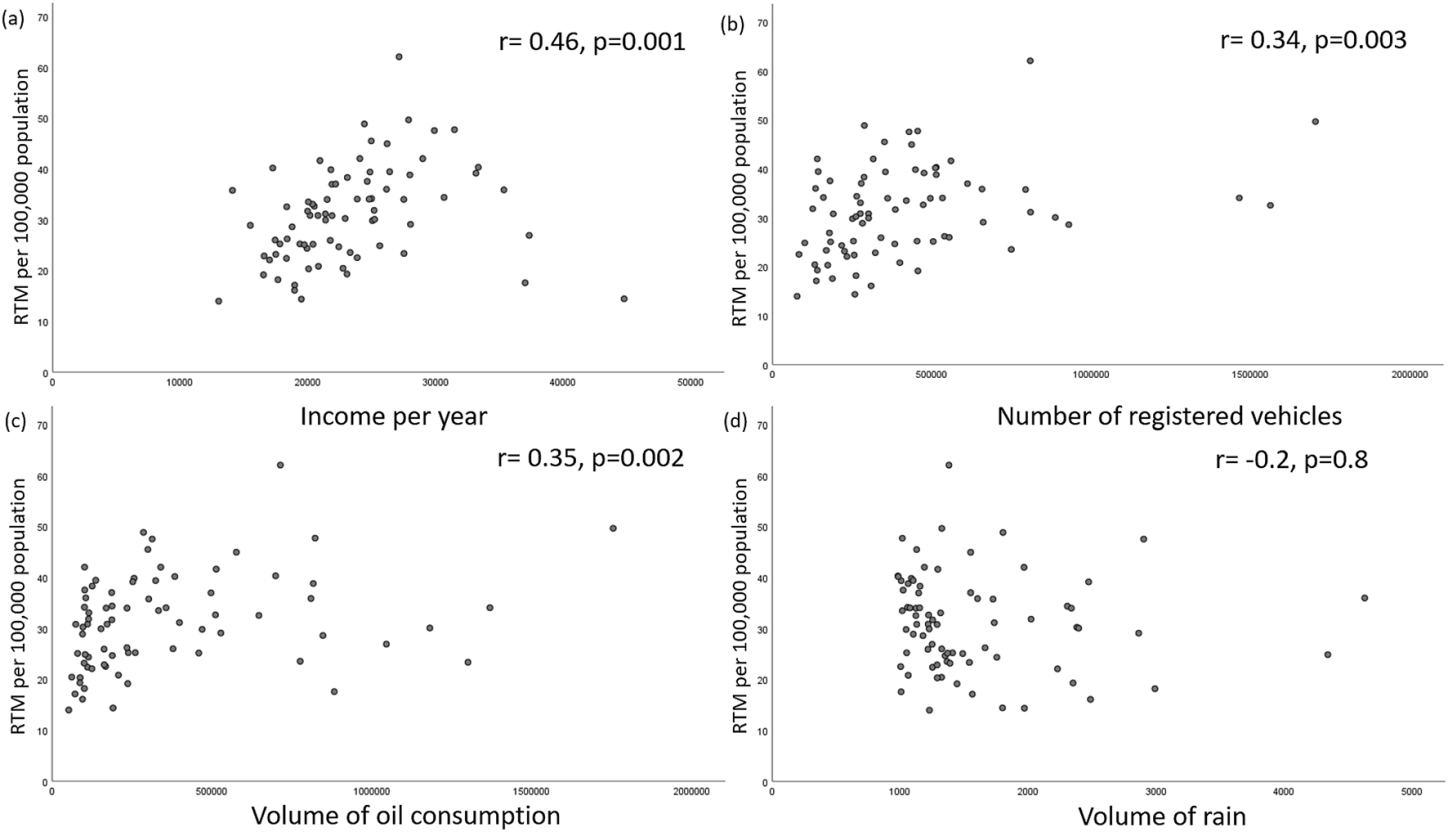
Correlations between road traffic mortalities (RTM) and (**a**) income per year, (**b**) number of registered vehicles, (**c**) volume of oil consumption, and (**d**) volume of rain in the 77 provinces of Thailand.

Income per year (P = 0.001), number of registered vehicles (P = 0.003), and amount of oil consumption (P = 0.002) all had moderate positive correlations with RTMs while the volume of rainfall did not (Table 4, Fig. 4).

**Table 4:** Correlations between road traffic mortality (RTM), and related factors during 2011–2021.

Related factors	RTM/100,000 person-year
Income per year	0.46 (0.001)
Number of registered vehicles	0.34 (0.003)
Volume of oil consumption	0.35 (0.002)
Volume of rain	−0.20 (0.8)

## Discussion

Thailand, part of Southeast Asia, is an LMIC with over 70 million people spread unevenly across 77 provinces, with half of registered vehicles being motorcycles. RTIs are the second leading cause of death in Thailand^16^, with approximately 30 out of 100,000 people dying from these incidents every year, and children accounting for over 10% of these fatalities. Throughout the 11 years of this study, motorcycles were the main vehicles involved in RTMs, followed by cars, and a significant difference was seen between mortality rates before and during the COVID-19 pandemic. There were significant disparities between the distribution of hospital resources and rates of RTMs across Thailand.

### Overall

In 2010, RTIs accounted for 334,815 deaths in South-East Asia. RTMs were higher in middle-income nations than in low-income countries^17^. Thailand had on average 30.34 deaths per 100,000 population from RTIs, and neighbouring LMIC Malaysia reported similar mortality rates of 34.5 RTMs per 100,000 population^18^. In contrast, mortality rates from RTIs in two other Southeast Asian countries, Laos and Vietnam, were 11.6^19^ and 20.3^20^ per 100,000 population respectively.

RTMs are a significant health concern in the paediatric population. Studies have analysed injury rates of children involved in road traffic accidents, and LMICs have been found to account for 95% of RTMs in children globally^21^. Our study showed that the mortality rate for children involved in RTIs was 19.08 per 100,000 and rose to as high as 26 per 100,000 children. Previous studies in Thailand have reported that 80% of the injured and dead from RTIs were motorcyclists^22^. Traffic accidents were the second most common paediatric injury in Thailand, with head injury being the most common cause of death^23^. For adolescents aged 14–19, road accidents were reported as the number one cause of death^24^. Malaysia reported that in 2013, the main cause of death for 10- to 24-year-old males was transport-related injury, with those sustained by people traveling by car and motorbike responsible for 20 and 5.5 per 100,000 deaths from all causes respectively^25^. In stark contrast, a study from Australia, a high-income country, showed RTI mortality rates at between 6.3 and 10.3 per 100,000 population^26^, and mortality rates for children < 15 years old in the United States ranged between 0.25 and 21.91 deaths per 100,000 children. Decreased mortality rates have been associated with the availability of trauma centers in the county^27^.

Premature childhood deaths result in societal as well as economic losses, with Thailand reporting that premature mortality contributed up to 88% of DALYs lost due to RTIs. This is high compared to other countries such as Australia (73%), Iran (62%), and Serbia (57%)^11^. Higher proportions of RTMs in LMICs result from the popularity of motorcycles, which are more affordable in these countries. Thailand was faced with a loss of about 100 million USD of quality-adjusted life years or approximately 300 million USD of value of statistical life years from traffic mortalities between 2010 and 2012^24^.

Lockdown policies during the COVID-19 pandemic led to a sharp drop in traffic volume and a global decline in RTIs^28,29^. Travel restrictions imposed during COVID-19 significantly reduced vehicle mobility by more than 50% worldwide. Though relative increases in severity of injury and numbers of deaths were observed, the pandemic reduced the absolute number of RTIs. Like other countries, Thailand faced lockdown policies and decreased road usage together with alcohol restrictions. These road usage restrictions were implemented in April 2020, when people were not allowed to leave their homes between 10 PM and 4 AM, and traveling between cities was prohibited. The attendant decrease in the number of hours of road usage, along with the alcohol-free period, led to a significant decline in RTMs in Thailand during the COVID-19 pandemic. Interestingly, in 2021, around 21,000 people died from COVID-19 infection, while deaths form traffic accidents numbered approximately 12,000.

The average age of people dying as a result of RTIs was 40 years old, which is in the working age group. Productivity losses due to road traffic accidents are mainly concentrated in the 16- to 45-year-old groups. Road traffic injuries and fatalities in young adults significantly affect the nation’s GDP since younger people bear the largest share of the economic burden^10,22^.

### Type of road accidents

Regions that were more populated did not necessarily have higher RTMs. Distribution of RTMs by road user groups has been shown to vary across countries. Motorcyclists account for most of the RTMs in Southeast Asian regions, while motorised four-wheelers constitute less than 20% of traffic-related mortalities^6^, and this in keeping with the findings of our study. Eighty-eight percent of motorized 2–3 wheelers are found in LMICs, with 75% in Southeast Asia^30^. Thailand was noted to have a high usage of 2-wheelers in addition to lax law enforcement, causing higher 2-wheeler deaths than in countries like Japan where law enforcement is more rigorous^4^. The second most common ICD-10 diagnosis from our study of persons injured in motor-vehicle accidents was specified as motorcycle injury. Similarly, Laos reported that 76% of RTIs involved motorcyclists^19^.

### RTMs across each province

Rayong and Chonburi were the two provinces with the highest RTMs at 62 and 49 per 100,000 population, possibly because they had the highest proportion of motorcycles per population; in fact, the number of registered motorcycles exceeded the population of the provinces. Bangkok has about a tenth of the country’s population with RTMs at only about a fourth of these top two provinces. This highlights the fact that higher numbers of people does not necessarily mean more road accidents. Studies have found that less-urbanised districts were associated with higher mortality than large metropolitan areas^27^. Other risk factors involved in RTMs in Southeast Asia were type of roads, number of male motorcycle drivers, driving without a driver’s license, and non-use of helmets^31^.

### Distribution of hospital resources

Both in-hospital and pre-hospital care have been identified as factors affecting RTMs^32–37^. A paper from Iran showed that pre-hospital trauma care was dispensed unequally across the nation and should be adjusted to reduce the number of RTMs^32^. The estimated number of lives that could potentially be saved globally if a complete trauma system with 100% coverage was available in LMICs was estimated at 200,000 per year. Having trauma centres and efficient trauma teams has also been shown to reduce deaths from RTIs^38^. Mortality rates from motorcycle injuries in the United Arab Emirates dropped significantly due to improved pre-hospital and in-hospital trauma care^39^.

Our study looked further into the distribution of available hospital resources and found that they were not balanced in accordance with mortality rates from RTIs in each province. A paper from Poland reported that poor HCRs were responsible for anomalies in mortality rates due to traffic accidents in each region^9^ and that ORs were found to be the least equally distributed out of all the hospital resources. This is due to the extensive process required to provide appropriate venues, as well as surgical teams and equipment, making it harder to open ORs. In contrast, physicians in Thailand were more equally distributed than other resources, and this could be because Thailand has been trying to allocate enough doctors to each province according to its population. HCRs were previously allocated according to the number of people in each province, which was why Bangkok, which had the highest population, had the most HCRs per 100,000 population. Physicians and nurses could be reallocated appropriately, and provision of other facilities, such as ORs and ICUs, could be integrated into healthcare policies, while taking into account the rates of RTMs in each province and focusing attention on trauma teams and facilities.

### Associated factors

Gross national income (GNI), urban speed limits, road quality, and regular road infrastructure inspections have all been shown to be influential factors in the rate of RTMs^40–44^. Countries with high GNI per capita have fewer deaths per 100,000 population even though they have higher numbers of vehicles while nations with low GNI per capita have higher rates of deaths per 100,000 population despite having fewer vehicles^4^.

Traffic injuries have repeatedly been shown to follow the trends of economic growth. Globally, a drastic increase in the number of vehicles led to RTMs reaching 135 cases per 100 vehicles in the year 2000, but this fell to 64 cases per 100 vehicles in 2016^31^. Other factors that were found from this study to correlate with RTMs were, income, number of registered vehicles, and amount of precipitation. RTIs in Thailand have been shown to go in the same direction as the nation’s economy^45^.

Amounts of precipitation did not correlate with RTMs in our study, in contrast to another study previously conducted in Thailand which found a significant increase in road accidents resulting from high rainfall. These contradictory results were probably due to differences in data collection and analysis: our study calculated the amount of rain throughout the year and found no correlation with RTMs while the previous study grouped different rain intensities measured by daily rates of precipitation^46^.

### Strengths and weaknesses

One of the main strengths of this study was that it provided the largest data available in all 77 provinces in Thailand from various reliable governmental resources and collected them over a period of 11 years. We also divided the population into adults and children so that Thailand could have data on different age groups, which will be helpful when allocating medical personnel, since children and adults require different types of medical attention.

Another strength of this study is that we showed which hospital resources were the most unequally distributed in order to help prioritise which resources needed to be adjusted first.

The limitations of this paper were that although we had the number of RTMs of each province, we did not know the exact location where the accidents occurred; therefore, we could not assess factors such as road types which have shown to be associated with RTMs. We also did not have details on levels of alcohol consumption or helmet usage in the reported RTMs, both of which affect mortality. We reported RTM rates for children but did not sub-group physicians into paediatricians and emergency department physicians, so that we could not establish how effectively trauma cases are handled in each province. The times at which the accidents occurred were not available, and we did not divide the period into weekdays, weekends, and long holidays, so we were not able to analyse these risk factors of RTMs, as this was not within the scope of the study.

## Conclusion

RTM distribution varies across the world and also across different areas within countries; therefore, effective policies and resource adjustments should be based on evidence for each individual nation. We have provided information regarding distribution of RTMs across all the provinces in Thailand, further dividing them into adults and children, who require different types of medical attention. A marked inequality was found between the distribution of RTMs and availability of hospital resources, and we hope that our report will be of use in allocating these resources more appropriately in order to cater for the amount of traffic accidents in each area and lower traffic-related deaths.

## Supplementary Information

Supplementary Table S1.

## Data Availability

The datasets used and/or analysed during the current study are available from the corresponding author on reasonable request.
