# Supplementary material for: Characteristics of road traffic mortality and distribution of healthcare resources in Thailand
Source: Sci Rep. 2022 Nov 24;12:20255. doi: 10.1038/s41598-022-24811-4 (PMC9686261; doi:10.1038/s41598-022-24811-4)
Supplement: Supplementary file 1 — Supplementary Table S1. [file 41598_2022_24811_MOESM1_ESM.docx]

| Ranking | Province | RTM/100000  total | RTM/100000  adult | RTM/100000  children | Bed/100000 | OR/100000 | ICU/100000 | Physician/100000 | Nurse/100000 | Population |
| --- | --- | --- | --- | --- | --- | --- | --- | --- | --- | --- |
| 1 | Rayong | 62.05 * | 66.94 * | 30.42 | 184.76 | 3.70 | 8.53 | 40.82 | 223.54 | 703628.20 |
| 2 | Chon Buri | 49.63 | 53.14 | 20.92 | 106.95 | 2.96 | 9.95 | 76.97 | 291.13 | 1486729.70 |
| 3 | Prachin Buri | 48.83 | 49.93 | 23.81 | 183.16 | 3.70 | 15.64 | 36.48 | 197.61 | 485913.40 |
| 4 | Saraburi | 47.70 | 45.81 | 37.00 | 215.29 | 4.07 | 11.74 | 52.78 | 264.97 | 638686.90 |
| 5 | Chanthaburi | 47.53 | 48.98 | 32.70 | 204.04 | 3.57 | 13.35 | 56.28 | 286.80 | 531769.40 |
| 6 | Pachuap kiri khan | 45.48 | 46.42 | 29.29 | 188.96 | 4.27 | 17.45 | 33.74 | 171.65 | 538744.00 |
| 7 | Chachoengsao | 44.94 | 46.19 | 26.16 | 165.71 | 3.54 | 8.07 | 35.98 | 180.18 | 706663.30 |
| 8 | Nakhon Nayok | 42.01 | 41.88 | 29.30 | 189.47 | 3.09 | 10.44 | 96.94 | 340.69 | 258619.40 |
| 9 | Chumphon | 42.00 | 41.08 | 29.52 | 187.62 | 3.16 | 11.07 | 31.18 | 197.76 | 505821.70 |
| 10 | Phitsanulok | 41.61 | 42.83 | 26.32 | 172.81 | 3.49 | 13.03 | 72.11 | 254.87 | 859327.00 |
| 11 | Nakhon Pathom | 40.30 | 40.78 | 20.09 | 145.96 | 2.87 | 7.19 | 49.40 | 194.98 | 904369.60 |
| 12 | Suphan Buri | 40.16 | 40.26 | 20.30 | 194.00 | 4.73 | 11.93 | 32.17 | 195.68 | 846373.80 |
| 13 | Lopburi | 39.82 | 35.02 | 25.42 | 170.19 | 3.05 | 4.51 | 36.66 | 202.89 | 754437.40 |
| 14 | Sing Buri | 39.42 | 34.31 | 20.76 | 272.59 | 5.72 | 15.73 | 41.32 | 400.64 | 209841.30 |
| 15 | Phet Buri | 39.36 | 38.56 | 24.47 | 171.18 | 3.76 | 13.78 | 30.94 | 215.89 | 479032.20 |
| 16 | Phuket | 39.13 | 40.31 | 22.68 | 172.33 | 5.06 | 14.17 | 83.05 | 298.38 | 395163.10 |
| 17 | Ayutthaya | 38.79 | 36.91 | 23.22 | 130.79 | 2.71 | 9.75 | 32.46 | 194.42 | 810483.90 |
| 18 | Lamphun | 38.29 | 39.70 | 14.71 | 167.00 | 3.21 | 10.38 | 35.28 | 217.92 | 404789.10 |
| 19 | Chai Nat | 37.52 | 39.07 | 16.73 | 169.41 | 3.65 | 8.52 | 29.23 | 220.09 | 328781.50 |
| 20 | Sa Kaeo | 37.01 | 36.99 | 30.93 | 134.34 | 2.69 | 7.70 | 21.78 | 133.65 | 558265.50 |
| 21 | Nakhon Sawan | 36.95 | 36.62 | 24.46 | 124.65 | 2.35 | 7.72 | 38.43 | 172.53 | 1062216.30 |
| 22 | Trat | 35.97 | 34.75 | 17.35 | 207.30 | 3.07 | 7.03 | 39.18 | 292.50 | 227690.00 |
| 23 | Surat Thani | 35.85 | 36.48 | 28.49 | 169.73 | 2.85 | 10.64 | 43.06 | 240.59 | 1052229.70 |
| 24 | Chiang Rai | 35.75 | 35.13 | 21.99 | 135.71 | 3.24 | 7.59 | 35.09 | 174.93 | 1264477.60 |
| 25 | Krabi | 34.37 | 35.55 | 26.06 | 133.32 | 1.93 | 5.15 | 24.04 | 158.18 | 465806.70 |
| 26 | Ang Thong | 34.13 | 33.26 | 24.56 | 222.08 | 8.19 | 9.97 | 35.59 | 252.15 | 280978.70 |
| 27 | Nakhon Ratchasima | 34.04 | 34.93 | 22.38 | 137.29 | 2.81 | 7.64 | 34.67 | 167.71 | 2629401.60 |
| 28 | Ratchaburi | 34.03 | 33.79 | 22.54 | 256.49 | 2.43 | 14.92 | 44.05 | 241.61 | 864364.30 |
| 29 | Trang | 33.97 | 35.56 | 23.02 | 157.06 | 2.97 | 5.31 | 36.79 | 212.79 | 639890.00 |
| 30 | Phetchabun | 33.96 | 34.30 | 22.69 | 133.38 | 2.82 | 10.59 | 21.20 | 132.39 | 991874.90 |
| 31 | Kanchanaburi | 33.48 | 30.48 | 25.30 | 157.43 | 3.54 | 9.59 | 28.07 | 156.78 | 875929.20 |
| 32 | Uttaradit | 33.05 | 30.95 | 15.56 | 195.13 | 3.29 | 12.28 | 38.75 | 225.88 | 456114.50 |
| 33 | Lampang | 32.63 | 33.44 | 11.50 | 173.22 | 5.24 | 14.23 | 48.07 | 256.27 | 744728.00 |
| 34 | Chiang Mai | 32.52 | 31.00 | 17.46 | 131.00 | 2.42 | 11.43 | 62.17 | 306.74 | 1732840.90 |
| 35 | Phung Nga | 31.82 | 30.12 | 22.72 | 231.81 | 5.29 | 9.82 | 33.98 | 262.92 | 264870.00 |
| 36 | Kamphaeng Phet | 31.67 | 31.27 | 23.28 | 125.44 | 2.62 | 3.58 | 19.71 | 133.70 | 725447.60 |
| 37 | Ubon Ratchathani | 31.12 | 33.60 | 21.87 | 155.92 | 3.34 | 15.55 | 32.82 | 187.66 | 1858604.90 |
| 38 | Phrae | 30.83 | 28.65 | 17.09 | 183.19 | 3.35 | 6.93 | 33.31 | 226.73 | 447623.00 |
| 39 | Phichit | 30.81 | 30.31 | 25.06 | 156.99 | 3.69 | 7.02 | 34.04 | 169.13 | 541449.00 |
| 40 | Uthai Thani | 30.77 | 28.39 | 41.72 * | 228.04 | 2.74 | 6.39 | 28.99 | 198.49 | 328884.70 |
| 41 | Phatthalung | 30.23 | 30.20 | 19.82 | 155.18 | 3.07 | 9.00 | 23.30 | 201.24 | 521976.20 |
| 42 | Songkhla | 30.05 | 31.40 | 19.05 | 132.01 | 3.11 | 10.60 | 65.00 | 300.32 | 1415075.50 |
| 43 | Loei | 29.91 | 29.57 | 24.34 | 150.47 | 2.35 | 5.64 | 24.03 | 173.15 | 637998.20 |
| 44 | Samut Sakhon | 29.79 | 27.95 | 17.16 | 153.06 | 3.59 | 12.93 | 5.72 ^ | 246.55 | 556644.90 |
| 45 | Nakhon Si Thammarat | 29.07 | 28.11 | 19.37 | 141.83 | 3.35 | 11.48 | 28.62 | 163.28 | 1551162.70 |
| 46 | Phayao | 28.87 | 27.46 | 13.63 | 169.75 | 2.72 | 7.54 | 32.59 | 247.67 | 477768.30 |
| 47 | Khonkaen | 28.58 | 29.10 | 17.71 | 143.76 | 3.23 | 7.63 | 71.84 | 267.61 | 1794635.80 |
| 48 | Pathum Thani | 26.89 | 24.72 | 18.76 | 51.92 ^ | 1.16 ^ | 5.19 | 64.29 | 173.31 | 1117196.10 |
| 49 | Sakon Nakhon | 26.19 | 27.13 | 17.73 | 170.64 | 4.02 | 10.05 | 21.55 | 146.70 | 1143915.70 |
| 50 | Buri Ram | 25.98 | 25.29 | 19.44 | 157.49 | 2.65 | 3.35 | 23.05 | 137.13 | 1583547.50 |
| 51 | Sukho Thai | 25.91 | 25.22 | 14.79 | 163.53 | 3.35 | 14.23 | 25.91 | 179.98 | 597460.00 |
| 52 | Tak | 25.22 | 23.36 | 16.31 | 178.58 | 4.38 | 10.07 | 28.90 | 168.76 | 615970.90 |
| 53 | Roi Et | 25.21 | 23.34 | 16.43 | 130.96 | 1.99 | 7.66 | 21.88 | 142.13 | 1305697.70 |
| 54 | Surin | 25.14 | 25.80 | 15.47 | 141.26 | 2.88 | 11.22 | 23.60 | 133.66 | 1390301.70 |
| 55 | Mukdahan | 25.06 | 25.96 | 16.20 | 151.89 | 4.01 | 8.60 | 24.85 | 182.41 | 348939.40 |
| 56 | Ranong | 24.86 | 20.10 | 21.71 | 202.58 | 4.80 | 7.46 | 29.45 | 247.26 | 187578.50 |
| 57 | Maha Sarakham | 24.64 | 24.18 | 13.74 | 130.48 | 3.24 | 7.52 | 26.36 | 156.96 | 958021.50 |
| 58 | Nong Khai | 24.34 | 20.88 | 26.79 | 160.07 | 3.28 | 12.92 | 29.57 | 174.79 | 518516.30 |
| 59 | Udon Thani | 23.52 | 21.81 | 18.53 | 137.90 | 1.46 | 7.88 | 29.77 | 155.61 | 1573639.40 |
| 60 | Samut Prakan | 23.33 | 20.98 | 9.39 | 78.53 | 1.69 | 8.01 | 38.42 | 146.71 | 1298948.20 |
| 61 | Yasothon | 23.16 | 22.53 | 15.75 | 133.72 | 2.97 | 6.69 | 21.23 | 160.26 | 538432.20 |
| 62 | Kalasin | 22.84 | 20.95 | 3.56 ^ | 150.53 | 2.95 | 4.17 | 20.36 | 145.38 | 983197.00 |
| 63 | Samut Songkhram | 22.51 | 22.58 | 8.74 | 217.09 | 4.65 | 8.27 | 31.27 | 266.06 | 193468.40 |
| 64 | Nan | 22.37 | 22.14 | 13.41 | 190.26 | 4.60 | 10.45 | 35.63 | 246.90 | 478293.90 |
| 65 | Nakhon Phanom | 22.06 | 21.09 | 13.23 | 116.70 | 3.21 | 5.87 | 18.45 | 152.28 | 715501.40 |
| 66 | Chaiyaphum | 20.81 | 19.64 | 17.58 | 140.42 | 2.56 | 3.00 ^ | 20.34 | 136.56 | 1134493.60 |
| 67 | Bueng Karn | 20.41 | 20.15 | 14.90 | 114.16 | 4.28 | 5.47 | 13.03 | 99.79 ^ | 420478.70 |
| 68 | Nong Bua Lam Phu | 20.30 | 17.60 | 8.36 | 80.05 | 1.96 | 5.89 | 17.81 | 115.76 | 509688.90 |
| 69 | Satun | 19.29 | 19.58 | 15.73 | 134.14 | 3.15 | 7.56 | 23.87 | 219.89 | 317567.30 |
| 70 | Si Sa Ket | 19.13 | 18.56 | 13.00 | 129.62 | 2.32 | 7.23 | 19.54 | 122.98 | 1465878.70 |
| 71 | Narathiwat | 18.17 | 20.40 | 17.72 | 126.84 | 1.65 | 5.58 | 23.21 | 217.17 | 789192.60 |
| 72 | Nonthaburi | 17.56 | 16.82 | 9.00 | 78.79 | 1.89 | 3.78 | 49.74 | 193.54 | 1218424.80 |
| 73 | Amnat Charoen | 17.10 | 16.02 | 10.44 | 143.43 | 3.45 | 9.03 | 20.13 | 157.33 | 376478.50 |
| 74 | Yala | 16.08 | 17.09 | 17.12 | 173.79 | 3.06 | 12.80 | 34.80 | 283.70 | 523614.80 |
| 75 | Bangkok | 14.41 | 16.39 | 5.74 | 572.32 * | 9.27 * | 33.05 * | 133.49 * | 479.43 * | 5657669.40 * |
| 76 | Pattani | 14.35 | 15.12 | 14.03 | 134.37 | 1.70 | 8.52 | 23.54 | 207.34 | 704019.60 |
| 77 | Mae Hong son | 13.95 ^ | 12.80 ^ | 11.29 | 156.75 | 5.55 | 8.87 | 28.37 | 185.80 | 270498.80 |
|  | Average | 30.34 | 32.71 | 19.08 | 164.11 | 3.37 | 9.46 | 37.34 | 207.65 | 65754755 |

**^*^**Maximum in any variable

^ Minimum in any variable

**Abbreviation**: RTM, road traffic mortality; bed, hospital bed; OR, operating room; ICU, intensive care unit bed

**Supplementary Table S1** Road traffic mortality rates and hospital resources per 100,000 population in 77 provinces in Thailand, during 2011-2021.
